# Xiaoyaosan Ameliorates Chronic Immobilization Stress-Induced Depression-Like Behaviors and Anorexia in Rats: The Role of the Nesfatin-1–Oxytocin–Proopiomelanocortin Neural Pathway in the Hypothalamus

**DOI:** 10.3389/fpsyt.2019.00910

**Published:** 2019-12-10

**Authors:** Qingyu Ma, Xiaojuan Li, Zhiyi Yan, Haiyan Jiao, Tingye Wang, Yajing Hou, Youming Jiang, Yueyun Liu, Jiaxu Chen

**Affiliations:** ^1^Formula-Pattern Research Center, School of Traditional Chinese Medicine, Jinan University, Guangzhou, China; ^2^School of Traditional Chinese Medicine, Beijing University of Chinese Medicine, Beijing, China

**Keywords:** Xiaoyaosan, chronic immobilization stress, depression, anorexia, nesfatin-1

## Abstract

**Background:** Chronic stress is an important risk factor for depression. The nesfatin-1 (NES1)–oxytocin (OT)–proopiomelanocortin (POMC) neural pathway, which is involved in the stress response, was recently shown to have an anorectic effect in the hypothalamus. Our previous study showed that Xiaoyaosan, a well-known antidepressant used in traditional Chinese medicine, effectively relieved appetite loss induced by chronic immobilization stress (CIS). However, whether Xiaoyaosan ameliorates depression-like behaviors and anorexia by regulating the NES1-OT-POMC neural pathway remains unclear.

**Objective:** To investigate whether the antidepressant-like and anti-anorexia effects of Xiaoyaosan are related to the NES1-OT-POMC neural pathway in the hypothalamus.

**Methods:** Rats were randomly divided into control, CIS, Xiaoyaosan treatment, and fluoxetine treatment groups. The rats in the CIS, Xiaoyaosan treatment, and fluoxetine treatment groups were subjected to CIS for 21 consecutive days, during which they were administered distilled water, a Xiaoyaosan decoction [3.854 g/(kg·d)] or fluoxetine [1.76 mg/(kg·d)], respectively, by gavage, and their body weights and food intake were monitored daily. The rats were subsequently subjected to the open field test and sucrose preference test. Then, the expression levels of corticosterone and NES1 in the serum and the expression levels of NES1, OT, POMC, and melanocortin-4 receptor (MC4R) in the hypothalamus were determined by real-time fluorescence quantitative polymerase chain reaction, Western blot analysis, and immunochemistry. Furthermore, immunofluorescence double staining was used to determine whether related proteins in the hypothalamic NES1-OT-POMC neural pathway were co-expressed.

**Results:** Compared to control rats, rats exposed to CIS exhibited gradually less food intake and lower body weights and significantly increased concentrations of NES1 in the serum and paraventricular nucleus. Moreover, the expression levels of POMC, OT, and MC4R in the hypothalamus were significantly higher in the CIS group than those in the control group. However, these changes were reversed by pretreatment with Xiaoyaosan and fluoxetine. Specifically, the expression levels of members of the NES1-OT-POMC neural pathway were lower in the Xiaoyaosan-treated group than in the CIS group.

**Conclusion:** Xiaoyaosan ameliorates CIS-induced depression-like behaviors and anorexia by regulating the NES1-OT-POMC neural pathway in the hypothalamus.

## Introduction

Chronic stress causes a series of pathological disease states, including psychiatric disorders, such as major depressive disorder and anxiety disorders (1), and eating disorders, such as anorexia and bulimia nervosa (2) (3). In addition, changes in appetite and weight are common diagnostic markers for major depressive disorder (4), and approximately 48% of adult depressed patients show depression-related loss of appetite (5). The induction of some anorexigenic peptides by stress has been associated with the pathogenesis of depression (6).

Nesfatin-1 (NES1), an appetite-suppressing neuropeptide derived from the precursor protein nucleobindin-2 (NUCB2), is widely expressed in the central nervous system (CNS) and peripheral tissues. NES1 can cross the blood–brain barrier freely and plays an important role in the regulation of the stress response (6–8). The functions of NES1 include the regulation of mood, feeding, water intake, glucose metabolism, visceral sensitivity, blood pressure, reproduction, and sleep (8). Clinical studies have shown that changes in NES1 levels in peripheral blood are closely related to body mass index (9) and can be used as a marker to predict the severity of some stress-related diseases (6, 8, 10). More importantly, NES1 levels are positively correlated with the degree of depression (11).

In the CNS, NES1 is mainly distributed in the hypothalamic arcuate nucleus (ARC), the paraventricular nucleus (PVN), the supraoptic nucleus, and the solitary tract nucleus (NTS) of the brainstem. NES1 coexists or is co-expressed with a variety of appetite-regulating neuropeptides, such as oxytocin (OT) and proopiomelanocortin (POMC) (12). OT is a neuropeptide produced by the neurons of the hypothalamic PVN (13). NES1 targets both magnocellular and parvocellular OT neurons and NES1 neurons. Further research has confirmed that the neural information conveyed from NES1 to OT is linked to the melanocortin pathway (14). POMC neurons, an essential component of the melanocortin pathway, are located mainly in the hypothalamic ARC and brainstem NTS, where OT receptors are expressed (14). OT neurons in the PVN project to the ARC or NTS, where they activate POMC neurons. POMC neurons in the ARC project their axons to neurons in the PVN, where they regulate melanocortin-4 receptor (MC4R) function through the release of an MC4R-specific ligand, thus forming the NES1-OT-POMC neural pathway in the hypothalamus (12, 14, 15). Furthermore, several lines of evidence have indicated that the anorexigenic action of NES1 is blocked by an antagonist of the OT receptor and that OT-induced anorexia is blocked by an MC4R antagonist (12). The activation of the hypothalamic NES1-OT-POMC neural pathway, in which the suppressor peptide NES1 plays a pivotal role, effectively suppresses appetite.

Xiaoyaosan, a well-known traditional Chinese medicine (TCM) formula (16), has been used to treat various diseases for hundreds of years. Xiaoyaosan is effective in treating liver qi stagnation and spleen deficiency, a syndrome that causes both depression-like symptoms and appetite loss (17, 18). Previous studies have shown that Xiaoyaosan exerts an antidepressant-like effect by regulating brain regions such as the hippocampus (19, 20), amygdala (21), locus coeruleus (22), and hypothalamus (23). More importantly, our previous studies showed that Xiaoyaosan effectively regulated the levels of food-related neuropeptides, such as neuropeptide Y (NPY), in the hypothalami of rats exposed to chronic immobilization stress (CIS) for 21 days and improved appetite (24, 25). However, whether Xiaoyaosan can improve depression-like behaviors and anorexia by adjusting NES1 and NES1-related neural pathways in the hypothalamus is unclear.

In the present study, we hypothesized that Xiaoyaosan treatment regulates the NES1-OT-POMC neural pathway in the hypothalamus and thus ameliorates CIS-induced depression-like behaviors and anorexia. We therefore sought to verify this hypothesis by observing the effects of Xiaoyaosan on the regulation of NES1 and NES1-related feeding-regulatory peptides in rats exposed to CIS for 21 days.

## Materials and Methods

### Animals

Forty-eight specific pathogen-free (SPF), adult, male Sprague–Dawley (SD) rats that were healthy and weighed 180~220 g (animal license number: SCXK (Beijing) 2011-0004) were provided by SPF (Beijing) Biotechnology Co., Ltd. The rats were housed in plastic cages in an animal room (clean grade) at the Beijing University of Chinese Medicine (temperature: 22 ± 2 °C; relative humidity: 35% ± 5%; and light conditions: 12 h of light (light, 7:00 to 19:00; dark, 19:00 to 7:00)), given free access to distilled water and fed a regular rodent diet (SPF grade, supplied by Beijing Keao Xieli Feed Co., Ltd., Beijing, China).

After 1 week of adaptation, the rats were randomly assigned to 4 groups of 12 rats each: the control group, CIS group, Xiaoyaosan treatment group, and fluoxetine treatment group. The experimental protocols applied in this study were approved by the Institutional Animal Care and Use Committee at the Beijing University of Chinese Medicine and performed in accordance with the Beijing Laboratory Animal Ethics and Welfare Guidelines (issued on 2006-01-01) to minimize animal suffering and animal use.

### CIS Procedure

The rats in all groups, except for those in the control group, were fixed on a special frame designed to immobilize the rats (26). The immobilization frame was composed of a wooden T-shaped binding platform with a 10-cm wide, 20-cm long, and 2.8-cm thick base. The upper end of the platform was 22 cm long and 6.6 cm wide with a small frame that fixed the head at the front end and had grooves at the upper end suitable for binding the limbs. The table had two adjustable adhesive straps to bind the chest and abdomen of the rat. The rats were fixed in the frame at random time points for 3 h every day for 21 consecutive days. During the CIS procedure, the body weights and food intake of each rat were recorded daily until the end of the experiment.

### Preparation of Xiaoyaosan

According to a previous publication (26), the Xiaoyaosan formula in the book *Taiping Huimin Heji Jufang* was selected; this formula included the following eight raw herbs: *Radix Bupleuri, Radix Angelicae Sinensis, Radix Paeoniae Alba, Rhizoma Atractylodis Macrocephalae, Poria, Radix Glycyrrhizae, Herba Menthae*, and *Rhizoma Zingiberis Recens* at a ratio of 6:6:6:6:6:3:2:2. These herbs were purchased from Beijing Tongrentang Group Co., Ltd. and verified by the Pharmacy of Guoyitang of the Beijing University of Traditional Chinese Medicine. The herbs were processed into a dry extract in the China–Japan Friendship Hospital (Beijing, China) in accordance with the Regulation on Processing of Traditional Chinese Medical Herbal Pieces of Beijing. One gram of the dry extract contained 3.18 g of crude drug, which was mixed with distilled water. Meanwhile, the Xiaoyaosan fingerprint was analyzed by high-performance liquid chromatography (HPLC) and matched the typical chromatogram of that shown in a previous study (27).

### Drug Administration

**Figure 1:**
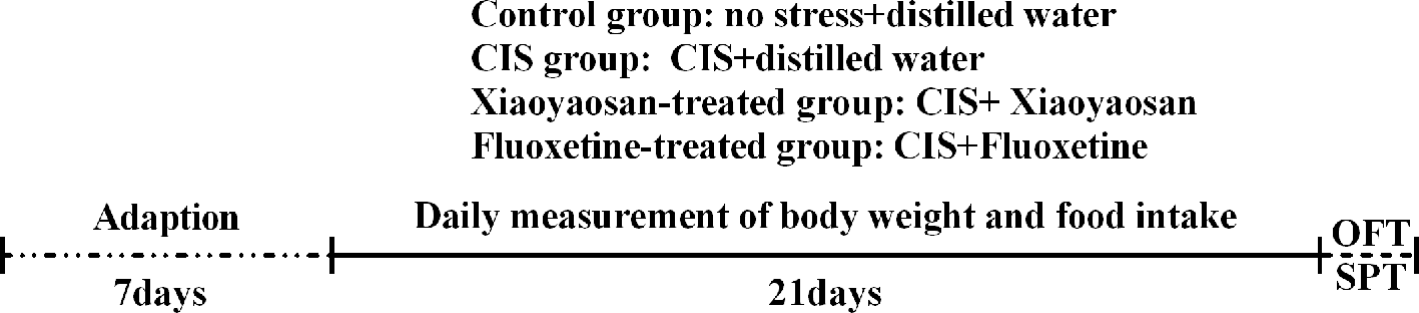
Study schedule. Before the experiment, the animals were allowed a 7-day adaptation period. Rats in all groups, except for those in the control group, were subjected to daily CIS for 3 h and administered the appropriate drug for 21 consecutive days. Body weights and food intake were recorded daily from the first day to the 21st day. The OFT was performed on day 20, and the SPT was performed on day 21. Rats were sacrificed on day 22.

Compounds were intragastrically administered by oral gavage using a straight ball-ended stainless-steel gavage needle attached to a 5-ml syringe to the corresponding group of rats 30 min before the start of CIS. Here, a conscious rat was manually immobilized, and the substance was administered slowly to avoid improper administration into the lungs. The drug dosage was determined according to the average adult body weight 70 kg/d dose conversion. The Xiaoyaosan treatment group was administered a Xiaoyaosan suspension at a dosage of 3.854 g/kg·d in 0.1 ml/kg of body weight, which was shown to be effective in our previous studies (26, 27). The fluoxetine treatment group was given fluoxetine (fluoxetine hydrochloride capsule, 20 mg/granule, dissolved in distilled water) at a dosage of 1.76 mg/kg·d in 0.1 ml/kg of body weight (28). The control group and the CIS group received 0.1 ml of distilled water/kg of body weight. The experimental design is shown in detail in **Figure 1**.

### Open Field Test (OFT)

The OFT was performed on the 20th day of the study as previously described (26). The open field box was placed in the middle of the operation room, and a camera connected to a computer and video recorder was placed directly above the middle box. Rats were placed in the dark and quiet environment of the operating room for 10 min before the experiment. An operator held the base of the tail of the rat and placed the rat in the middle of the box, and locomotor activity was then tracked using an electronic video tracking system (Noldus EthoVision, version 3.0). After 5 min, the rats were removed, the bottom of the box was thoroughly wiped with a towel dipped in water and a low concentration of alcohol, and the next rat was observed. After the experiment was completed, the total distance traveled and the time spent in the central area were calculated.

### Sucrose Preference Test (SPT)

The SPT was performed on the 21st day of the experimental process based on a previously described procedure (25). Briefly, the rats were fasted for 24 h before the test and then placed in a single cage at the same time of day. Two identical drinking bottles were weighed and marked in advance. The rats were free to ingest a 1% sucrose solution or distilled water. After 1 h, the two drinking bottles were simultaneously removed and weighed again. The sucrose preference of each rat, which is an objective indicator of hedonic behavior, was calculated.

### Double-Labeling Immunofluorescence Staining and Immunohistochemical Analysis

Brain tissues from the rats were fixed in 4% paraformaldehyde for 12 h and sliced into 4-µm-thick coronal sections. For double-label fluorescence staining of NES1 and POMC (or NES1 and OT), the sections were washed with phosphate-buffered saline (PBS), subjected to antigen retrieval by steam treatment for 5 min in 0.01 M sodium citrate (pH 6.0) and then blocked for 30 min at room temperature (RT) in 5% normal goat serum (ZSGB, Beijing, China). Then, the sections were incubated in 0.1% Triton X-100 in PBS with 0.1% Tween-20 (PBST) at RT for 30 min and blocked in 5% normal goat serum (ZSGB) in PBS at RT for 1 h. After the sections were washed with PBS, they were incubated with primary antibodies against both NES1 and POMC (or OT) at RT for 30 min to overnight. The sections were then incubated with two corresponding secondary antibodies (Alexa Fluor^®^ 488 goat anti-rabbit IgG H + L and Alexa Fluor^®^ 594 goat anti-mouse IgG H + L), and the cellular nuclei were counterstained with 4′, 6-diamidino-2-phenylindole (DAPI; Thermo Fisher Scientific, Waltham, MA, USA). Fluorescence images were acquired under an Olympus fluorescence microscope (BX53, Olympus Co., Tokyo, Japan), and fluorescence images of double-labeled cells were visualized by the microscope’s software to determine the co-expression status of NES1 and POMC (OT) in the hypothalamus.

For immunohistochemical analysis of NES1, POMC, and OT in the hypothalamus, the sections were washed with PBST, subjected to antigen retrieval, and then blocked for 30 min at RT in 5% normal goat serum. Sections were then incubated with the appropriate primary antibody at 4 °C overnight. After the sections were washed with PBST, they were incubated with corresponding secondary antibodies (ZSGB-BIO, Beijing, China) and treated with a 3,3′-diaminobenzidine tetrahydrochloride solution (ZSGB-BIO) for color development. The area of interest and the integral optical density were observed under a light microscope (BX53, Olympus Co.), and images were analyzed with Image-Pro Plus 6.0 software to obtain the mean optical density (MOD). The antibodies used in the present study are listed in **Table 2**.

### Enzyme-Linked Immunosorbent Assay

After blood was collected from the abdominal aorta, ELISA was used to determine the serum concentrations of corticosterone (CORT) (ADI-900-097, Enzo Life Sciences, New York) and NES1 (Cat. No. EK-003-22, Phoenix Pharmaceuticals, Inc., CA) according to the manufacturers’ protocols. All samples were analyzed in triplicate. For CORT, the sensitivity of the assay was 26.99 pg/ml, and the intra-assay and inter-assay coefficients of variation were 6.0% and 10.1%, respectively. For NES1, the limit of assay sensitivity was 1.26 ng/ml, and the intra- and inter-assay coefficients of variation were 8.6% and 13.1%, respectively. Although this ELISA kit is able to detect both NES1 and NUCB2, many studies have used it despite its cross-reactivity (29). The MOD at a wavelength of 450 nm was determined using a Multiskan™ GO (Thermo Fisher Scientific, Waltham, USA) detector system.

### Real-Time Fluorescence Quantitative Polymerase Chain Reaction (RT-qPCR)

The expression of NES1, OT, POMC, and MC4R mRNA in the hypothalamus was detected by RT-qPCR. Total RNA was extracted from hypothalamic tissues by TRIzol (Invitrogen, USA) and then subjected to 1% agarose gel electrophoresis to determine RNA integrity and contamination. The concentration of the total RNA was measured with a spectrophotometer (Eppendorf, Germany). Reverse transcription to synthesize cDNA was carried out according to the instructions of a RevertAid First Strand cDNA Synthesis Kit (Thermo Scientific, Waltham, MA) on a Mastercycler Gradient thermal cycler (Eppendorf, Germany) according to the manufacturer’s instructions. Primers were synthesized by Sangon Biotech Co., Ltd. (Shanghai, China), the sequences of which are shown in **Table 1**. Peptidylprolyl isomerase A/cyclophilin A (PPIA) was used as the housekeeping gene in this study. Fluorescence qPCR was performed using Power SYBR^®^ Green PCR Master Mix (Thermo Fisher Scientific) in a total volume of 20 μl by a CFX96™ Real-Time System (Bio-Rad, USA) with the following cycling parameters: 95 °C for 10 min and 40 cycles of 95 °C for 15 s and 55 °C for 60 s. Relative quantitative analysis of RT-qPCR results was performed by Bio-Rad CFX Manager 2.1 (Bio-Rad, USA).

**Table 1:** Primer sequences used in RT-qPCR analysis.

Gene		Sequences
PPIA	Forward	5′-GCATACAGGTCCTGGCATCT-3′
	Reverse	5′-CTTCCCAAAGACCACATGCT-3′
NES1	Forward	5′-AGTGAGGACGAGACTGGATGA-3′
	Reverse	5′-TGGTGGTTCAGGTGTTCAAA-3′
POMC	Forward	5′-CTATCGGGTGGAGCACTTCC-3′
	Reverse	5′-CGTTCTTGATGATGGCGTTC-3′
OT	Forward	5′-GCTGCGCTAGACCTGGATA-3′
	Reverse	5′-GGGCAGGTAGTTCTCCTCCT-3′
MC4R	Forward	5′-CCCGAGGTGTTTGTGACTCT-3′
	Reverse	5′-ATGGTTTCTGACCCGTTCG-3′

### Western Blot (WB) Analysis

The expression levels of the NES1, POMC, and MC4R proteins in the hypothalamus were analyzed by WB analysis. Total protein was extracted from the hypothalamus of each rat using a Tissue Protein Extraction Kit (CWBIO, Beijing, China), and a BCA protein assay kit (CWBIO) was used to determine the protein concentration. Proteins were separated through 12% SDS-PAGE gels and then transferred to polyvinylidene fluoride membranes. After the membranes were blocked with 5% skim milk in TBST for 30 min, they were incubated with primary antibodies overnight at 4 °C. After washing with TBST buffer, the membranes were incubated with the appropriate horseradish peroxidase-conjugated secondary antibodies. Then, bands corresponding to the proteins of interest were visualized by enhanced chemiluminescence reagent (Millipore, Billerica, MA, USA) and subsequently scanned and analyzed with an image analyzer (Bio-Rad, California, USA). The intensities of the protein bands were normalized to glyceraldehyde-3-phosphate dehydrogenase (GAPDH) intensity. The antibodies used in the present study are listed in **Table 2**.

**Table 2:** Antibodies used in this study.

Antigen	Host	Manufacturer	Cat. No.	Application	Dilution
NES1	Rabbit	Phoenix	H-003-22	WB	1:1,000
				IHC	1:2,000
				IF	1:500
POMC	Mouse	Abnoa	MAB11358	WB	1:1,000
				IHC	1:400
				IF	1:200
OT	Mouse	Merck	MAB5296	IHC	1:5,000
				IF	1:600
MC4R	Rabbit	Abcam	Ab24233	WB	1:300
GAPDH	Rabbit	TDYbio	TDY052F	WB	1:5,000
Alexa Fluor^®^ 488	Goat	Abcam	ab150077	IF	1:200
Alexa Fluor^®^ 594	Goat	Abcam	ab150116	IF	1:200
Goat anti-mouse IgG (H + L)	Goat	ZSGB-BIO	ZB-2305	WB	1:5,000
Goat anti-rabbit IgG (H + L)	Goat	ZSGB-BIO	ZB-2010	WB	1:7,000

### Statistical Analysis

All statistical analyses were performed using SPSS 21.0 software. All data are presented as the means ± SEMs. When general data were compared among groups, the normality of the data distribution and homogeneity of the data were first tested. If the data were normally distributed and homogenous, one-way ANOVA was used for statistical analysis. The least significant difference (LSD) method was used for comparisons between groups. If the data were not normally distributed or homogeneous, a non-parametric test was used. Multiple independent sample tests were performed for statistical purposes. A *P*-value <0.05 indicated significant differences for all the statistical tests. Data measured repeatedly (body weight, food intake) were statistically analyzed by ANOVA (first spherical test) with SPSS software using the general linear mode, and then the data at each time point were analyzed by one-way ANOVA.

## Results

### Xiaoyaosan Ameliorates CIS-Induced Depression-Like Behaviors in Rats

**Figure 2:**
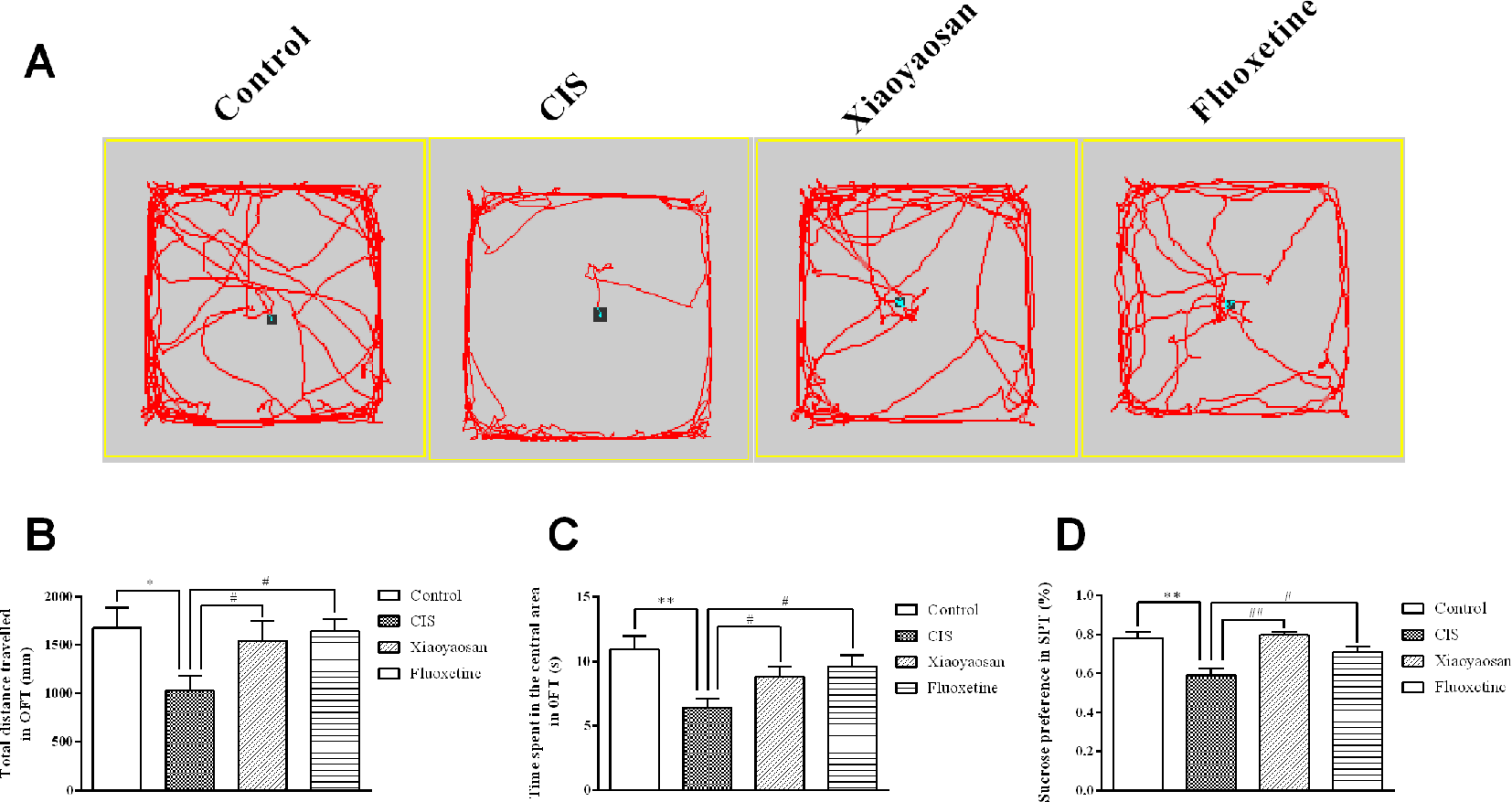
Xiaoyaosan ameliorates CIS-induced depression-like behaviors. **(A)** The paths taken by rats in each group in the OFT assessed by video tracking software. **(B)** Total distance traveled in 5 min in the OFT. **(C)** Time spent in the central area in the OFT. **(D)** Sucrose preference in the SPT. All the data are expressed as the mean ± SEM. ^*^P < 0.05, ^**^P < 0.01 compared with the control group; ^#^*P* < 0.05, ^##^*P* < 0.01 compared with the CIS group. *N* = 12 per group.

Before the experiment, the rats were allowed a 7-day adaptation period. The rats in all groups, except for those in the control group, were exposed to daily CIS for 3 h and administered the appropriate drug for 21 consecutive days. To evaluate the effects of 21-day CIS on the emotional function of rats and the regulation of these effects by Xiaoyaosan, the OFT was performed on day 20, and the SPT was performed on day 21 (**Figure 1**). We observed the total distance travelled and the time spent in the central area in the open field over a 5-min period and sucrose preference in the SPT. As shown in **Figures 2A–C**, the rats in the CIS group travelled a shorter total distance and spent less time in the central area over 5 min than those in the control group (*P* < 0.05, *P* < 0.01, respectively). These changes were effectively reversed by treatment with Xiaoyaosan (total distance traveled: *P* < 0.05 and time spent in the central area: *P* < 0.05) and fluoxetine (total distance traveled: *P* < 0.05 and time spent in the central area: *P* < 0.05). The SPT (**Figure 2D**) showed that the preference for sucrose of rats in the CIS group was significantly decreased compared with that of rats in the control group (*P* < 0.01). Compared with the rats in the CIS group, rats in the Xiaoyaosan and fluoxetine groups exhibited a significantly reversed sucrose preference (*P* < 0.01, *P* < 0.05). These results indicate that Xiaoyaosan significantly ameliorates depression-like behaviors after CIS.

### Xiaoyaosan Ameliorates CIS-Induced Anorexia in Rats

**Figure 3:**
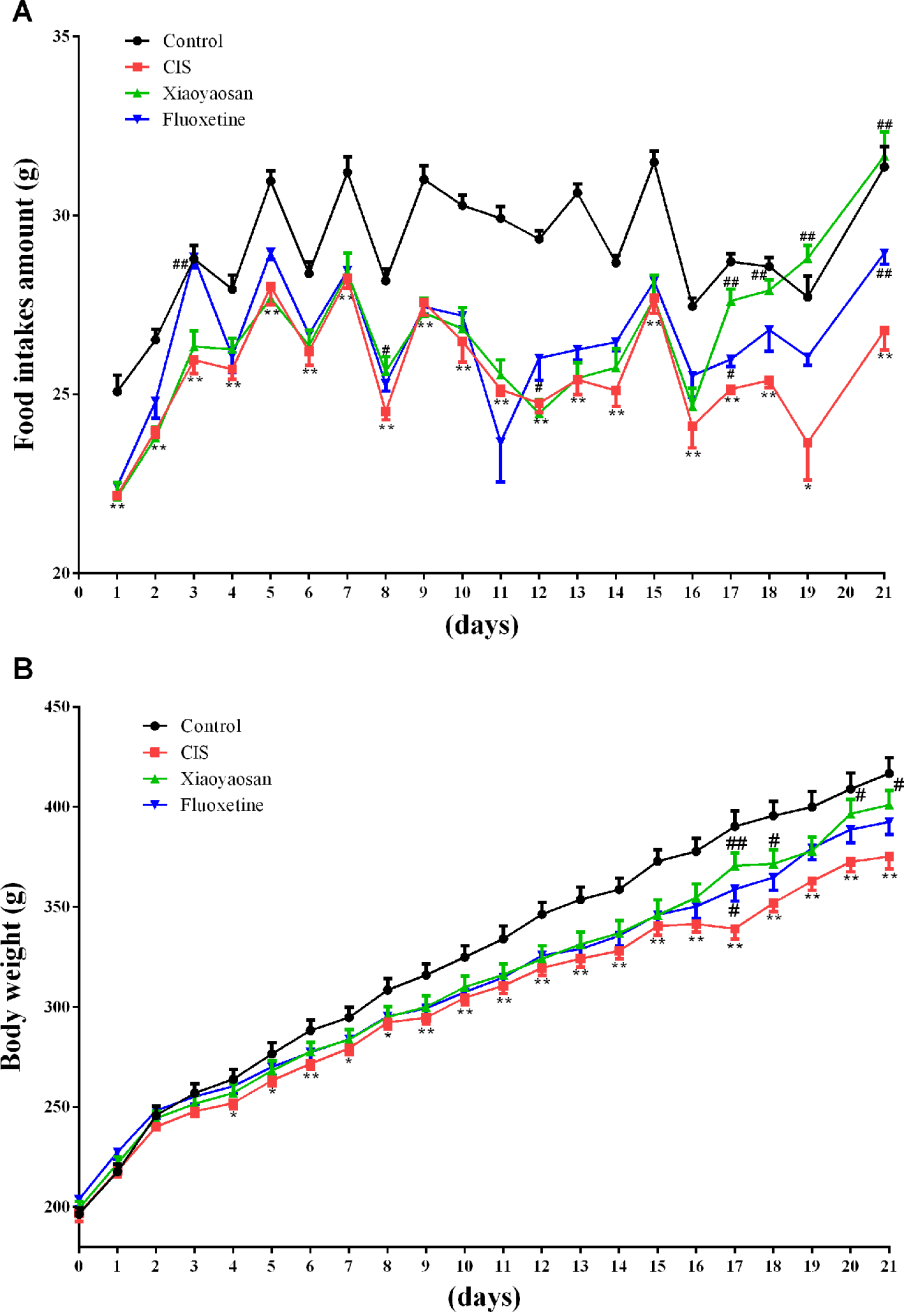
Xiaoyaosan ameliorates CIS-induced anorexia. **(A)** Changes in food intake. **(B)** Changes in body weight. All the data are expressed as the mean ± SEM. ^*^P < 0.05, ^**^P < 0.01 compared with the control group; ^#^*P* < 0.05, ^##^*P* < 0.01 compared with the CIS group. *N* = 12 per group.

Body weights and food intake were also recorded daily from the first day to the 21st day, to evaluate the effects of Xiaoyaosan on CIS-induced anorexia (**Figure 1**). Food intake data were not recorded for day 20 because all rats were fasted for 24 h before the SPT, which was performed on day 21. Food intake was affected by CIS, Xiaoyaosan treatment, and fluoxetine treatment (*P* < 0.05). As shown in **Figure 3A**, before CIS, there was no significant difference in food intake among the 4 groups of rats. On the 2nd day of CIS, rats in the CIS group consumed significantly less food than rats in the control group at the same time point (*P* < 0.05 or *P* < 0.01). However, from the 17th day to the 21st day of CIS, rats in the Xiaoyaosan group consumed significantly more food than rats in the CIS group (*P* < 0.01). **Figure 3B** shows that before CIS, there was no significant difference in body weight among the 4 groups of rats. On the 4th day of CIS, the rats weighed significantly less than the rats in the control group at the same time point (*P* < 0.05 or *P* < 0.01). However, from the 17th day to the 21st day, rats in the Xiaoyaosan group weighed significantly more than rats in the CIS group at the same time point (*P* < 0.05 or *P* < 0.01).

### Effects of Xiaoyaosan on Serum CORT and NES1 Levels in Rats After CIS

**Figure 4:**
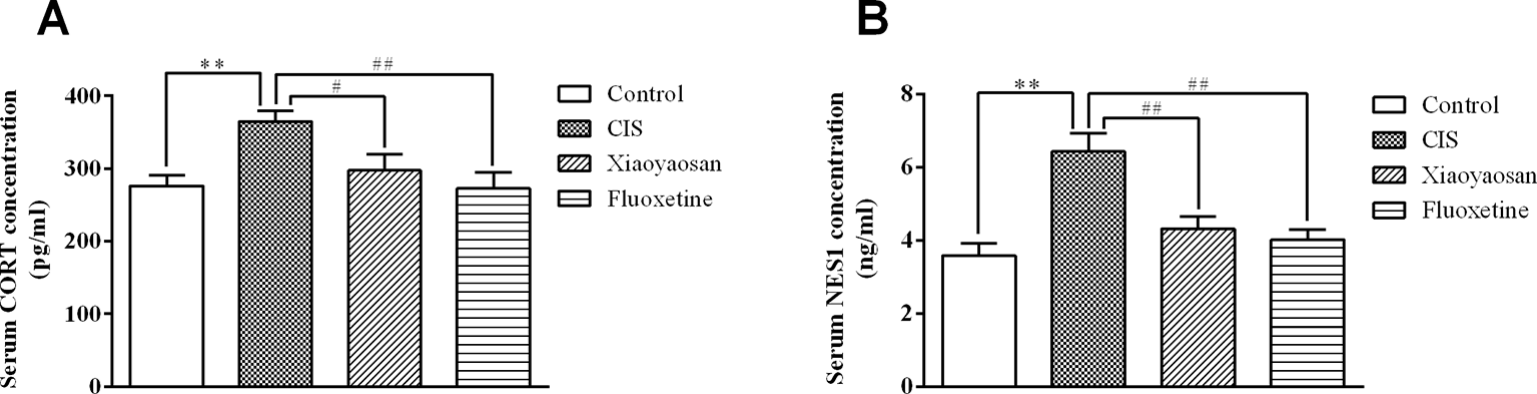
Effects of Xiaoyaosan on serum CORT and NES1 levels in rats after CIS. **(A)** Serum CORT concentration. **(B)** Serum NES1 concentration. All the data are expressed as the mean ± SEM from independent experiments performed in triplicate. ^**^ *P* < 0.01 compared with the control group; ^#^*P* < 0.05, ^##^*P* < 0.01 compared with the CIS group. *N* = 12 per group.

To detect the effects of Xiaoyaosan on stress-related signaling molecules, we sacrificed rats on day 22 (**Figure 1**) and determined the serum concentrations of CORT and NES1. As shown in **Figure 4A**, the serum CORT level in the CIS group were higher than those in the control group (*P* < 0.01), and Xiaoyaosan and fluoxetine treatment significantly reduced the CORT levels (*P* < 0.05 and *P* < 0.01, respectively). As shown in **Figure 4B**, the NES1 level in the CIS group was significantly higher than that in the control group (*P* < 0.01), and both Xiaoyaosan and fluoxetine significantly inhibited this increase in NES1 (*P* < 0.01 and *P* < 0.01, respectively) compared with CIS alone.

### Effects of Xiaoyaosan on the NES1-OT-POMC Neural Pathway in the Hypothalami of CIS-Induced Rats

**Figure 5:**
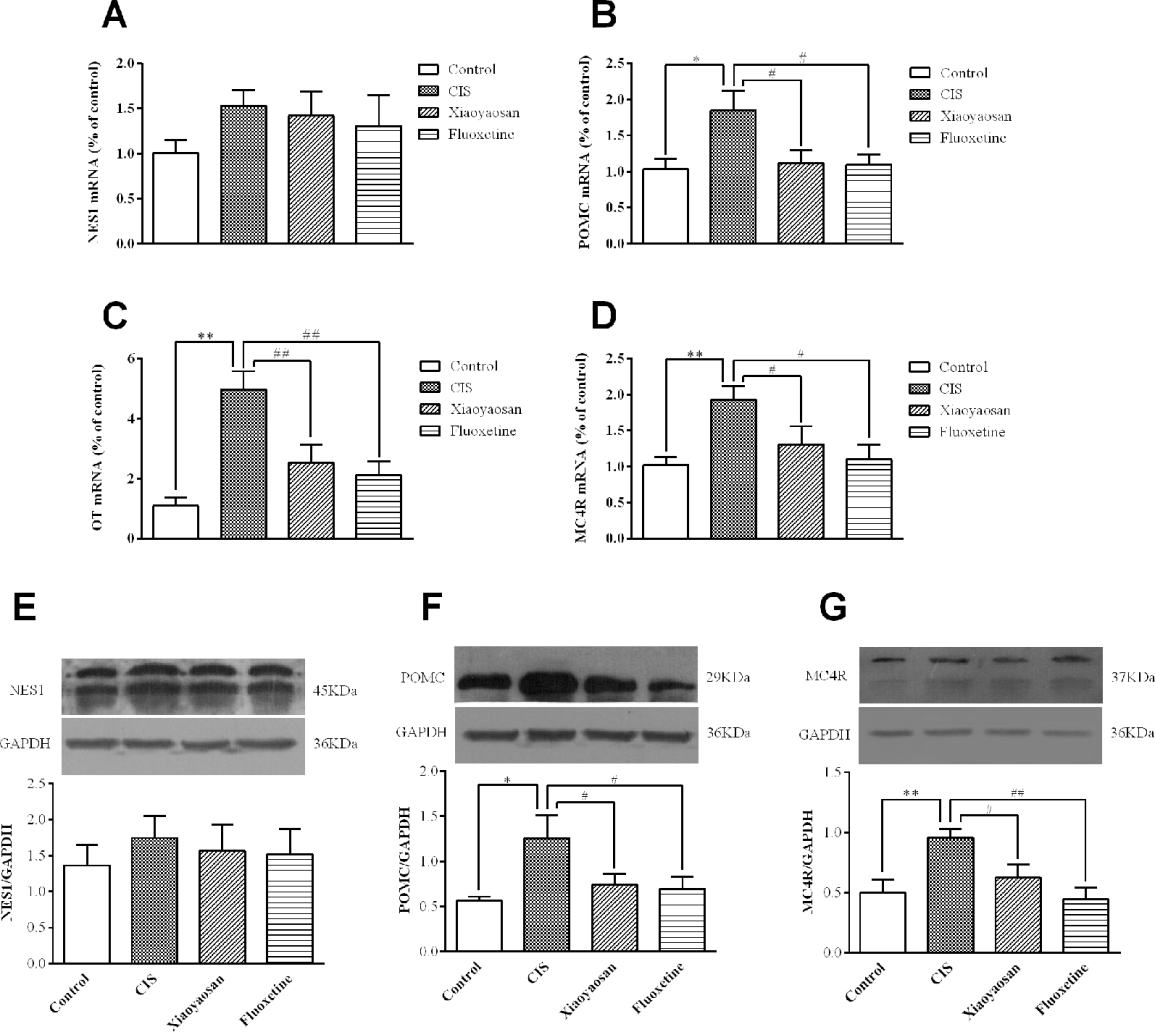
Effects of Xiaoyaosan on the NES1-OT-POMC neural pathway in the hypothalamus. mRNA expression levels were measured by RT-qPCR, and protein expression levels were determined by WB analysis. **(A)** NES1 mRNA expression (*n* = 6). **(B)** POMC mRNA expression (*n* = 6). **(C)** OT mRNA expression (*n* = 6). **(D)** MC4R mRNA expression (*n* = 6). **(E)** The protein expression of NES1 (*n* = 4). **(F)** The protein expression of POMC (*n* = 4). **(G)** The protein expression of MC4R (*n* = 4). All the data are expressed as the mean ± SEM from independent experiments performed in triplicate. ^*^P < 0.05, ^**^P < 0.01 compared with the control group; ^#^*P* < 0.05, ^##^*P* < 0.01 compared with the CIS group.

To validate our hypothesis, we examined the effects of Xiaoyaosan on the gene and protein expression of members of the NES1-OT-POMC neural pathway in the hypothalami of CIS-induced rats. As shown in **Figures 5A–D**, compared with those in the control group, the expression levels of POMC, OT, and MC4R mRNA in the hypothalami of rats in the CIS group were higher (*P* < 0.05, *P* < 0.01, and *P* < 0.01, respectively). This increase in the mRNA levels of POMC, OT, and MC4R was downregulated by Xiaoyaosan (*P* < 0.05, *P* < 0.01, and *P* < 0.05, respectively) and fluoxetine (*P* < 0.05, *P* < 0.01, and *P* < 0.05, respectively) treatment. NES1 mRNA levels showed a similar increase with CIS exposure, but the difference between NES1 mRNA levels was not significant (*P* > 0.05, **Figure 5A**). Changes in protein expression, which are shown in **Figures 5E–G**, exhibited the same trend. Whole gel picture of Western blotting is supplied in **Figures S1–S3**.

**Figure 6:**
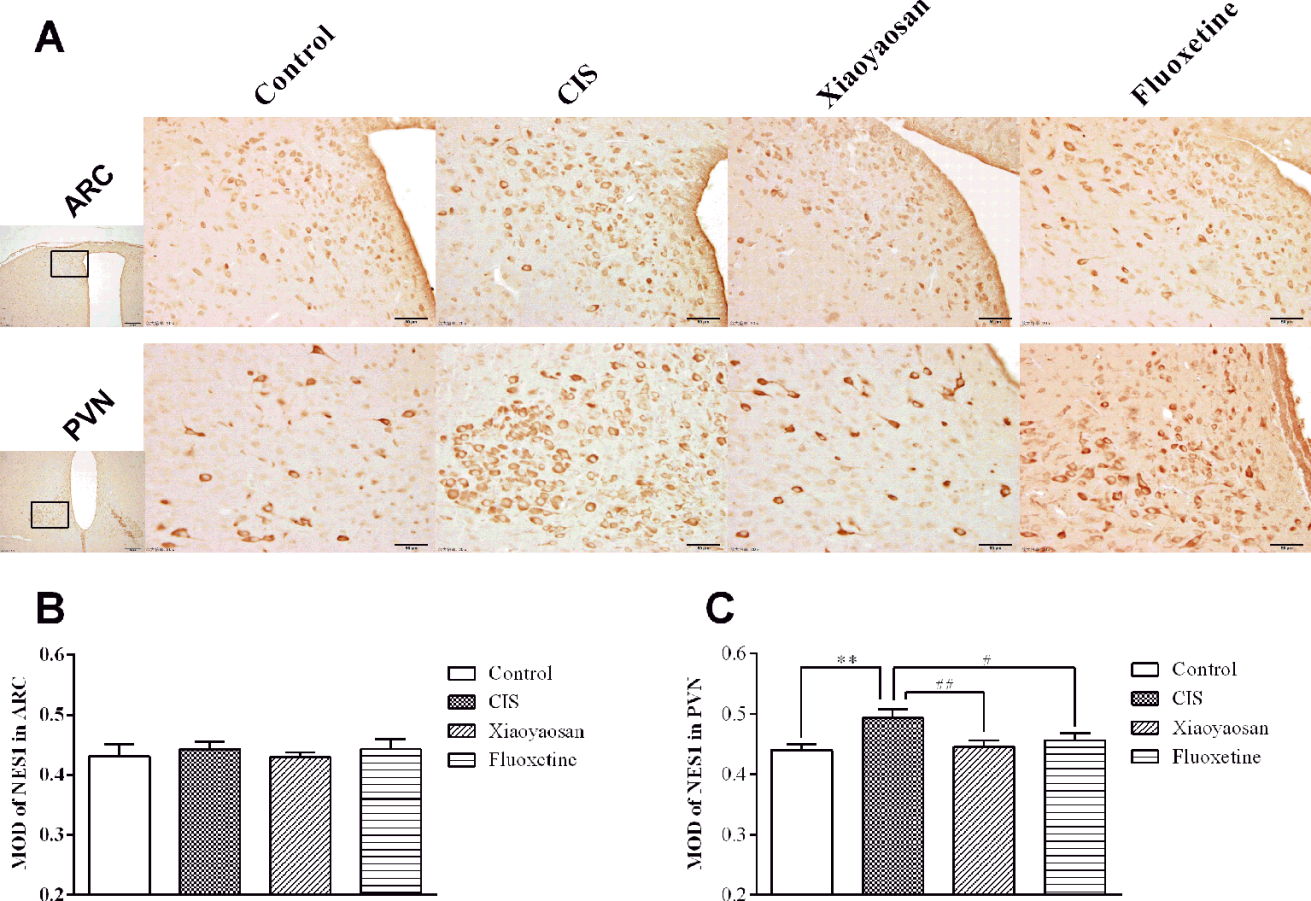
Effect of Xiaoyaosan on NES1 immunolabeling in the hypothalamus. **(A)** Representative micrographs showing immunohistochemical staining for NES1 protein in the ARC and PVN regions of the hypothalamus. Micrographs in the first column were captured at low magnification under a light microscope (scale bar = 200 μm, 100× magnification); the remaining micrographs were captured at high magnification under a light microscope (scale bar = 50 μm, 400× magnification). **(B)** Quantitative analysis of NES1 levels in the ARC. **(C)** Quantitative analysis of NES1 levels in the PVN. All the data are expressed as the mean ± SEM from independent experiments performed in triplicate. ^**^P < 0.01 compared with the control group; ^#^*P* < 0.05, ^##^*P* < 0.01 compared with the CIS group. *N* = 6 per group.

**Figure 7:**
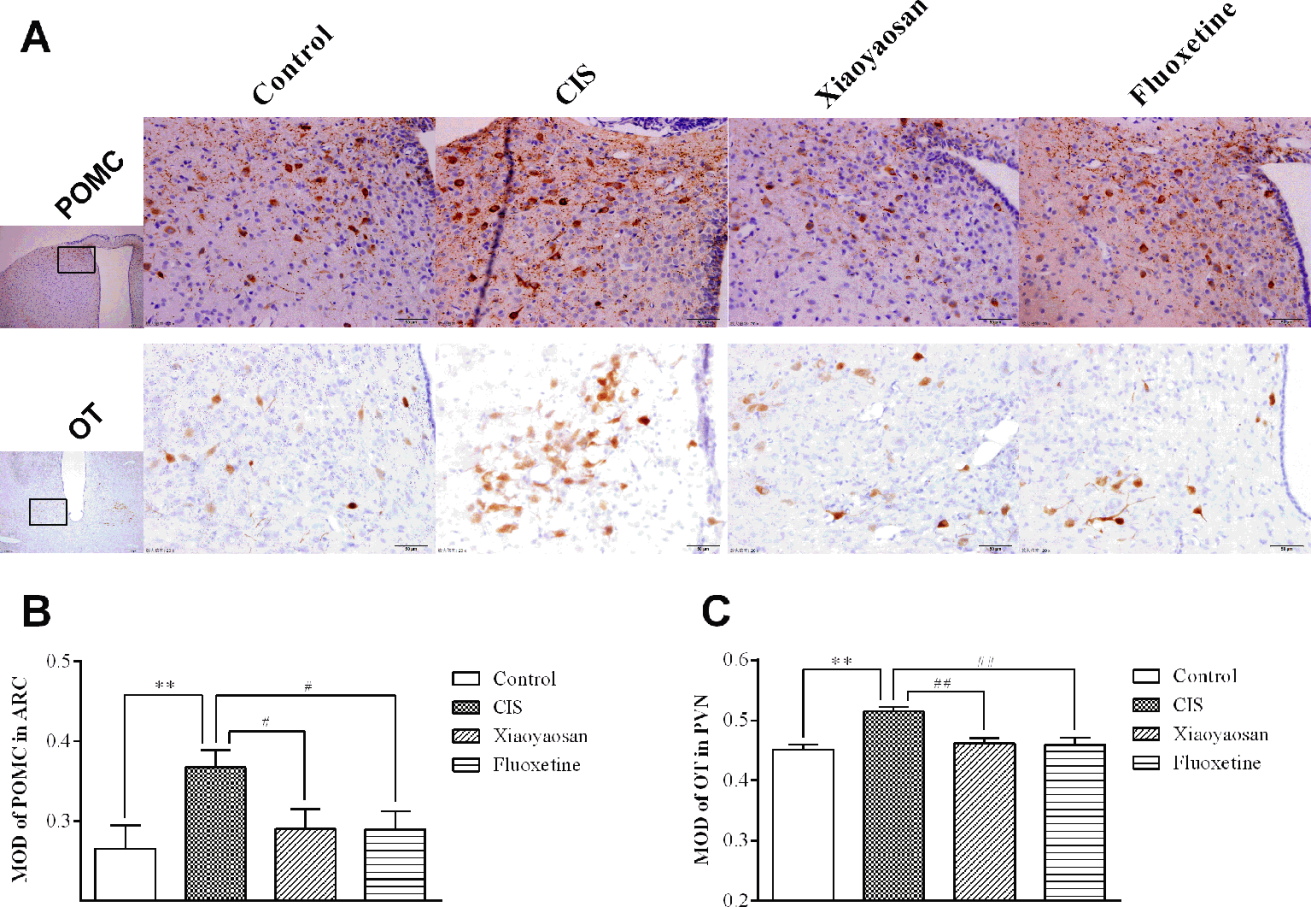
Effects of Xiaoyaosan on POMC and OT immunolabeling in the hypothalamus. **(A)** Representative micrographs showing immunohistochemical staining for the POMC and OT proteins in the hypothalamus. Micrographs in the first row show POMC protein in the ARC, and micrographs in the second row show OT protein in the PVN. Micrographs in the first column were captured at low magnification under a light microscope (scale bar = 200 μm, 100× magnification); the remaining micrographs were captured at high magnification under a light microscope (scale bar = 50 μm, 400× magnification). **(B)** Quantitative analysis of POMC levels in the ARC. **(C)** Quantitative analysis of OT levels in the PVN. All the data are expressed as the mean ± SEM from independent experiments performed in triplicate. ^**^P < 0.01 compared with the control group; ^#^*P* < 0.05, ^##^*P* < 0.01 compared with the CIS group. *N* = 6 per group.

As determined by immunohistochemistry experiments and shown in **Figures 6A** and 7A, B, NES1-, POMC-, and OT-immunopositive brown granules were observed in the hypothalamus. NES1-positive staining was widely distributed in the hypothalamic ARC and PVN. POMC-positive staining was mainly observed in the ARC, and OT-positive staining was distributed in the PVN. As shown in **Figure 6C**, the level of NES1 in the PVN of the hypothalami from rats in the CIS group was markedly higher than that in the control group (*P* < 0.01), whereas the levels of NES1 in the Xiaoyaosan group and fluoxetine group were significantly lower than those in the CIS group (*P* < 0.01 and *P* < 0.05, respectively). However, there was no significant difference in the level of NES1 in the hypothalamic ARC among the groups (both *P* > 0.05, **Figure 6B**). Moreover, as shown in **Figures 7C, D**, a significant increase in the levels of POMC in the ARC and OT in the PVN was observed in CIS rats compared to those in control rats (*P* < 0.01 and *P* < 0.01, respectively). After intervention with Xiaoyaosan and fluoxetine, the levels of POMC and OT were downregulated (POMC: *P* < 0.05 and *P* < 0.05, respectively; OT: *P* < 0.01 and *P* < 0.01, respectively).

### Co-Expression of NES1 and POMC (or OT) in the Rat Hypothalamus

**Figure 8:**
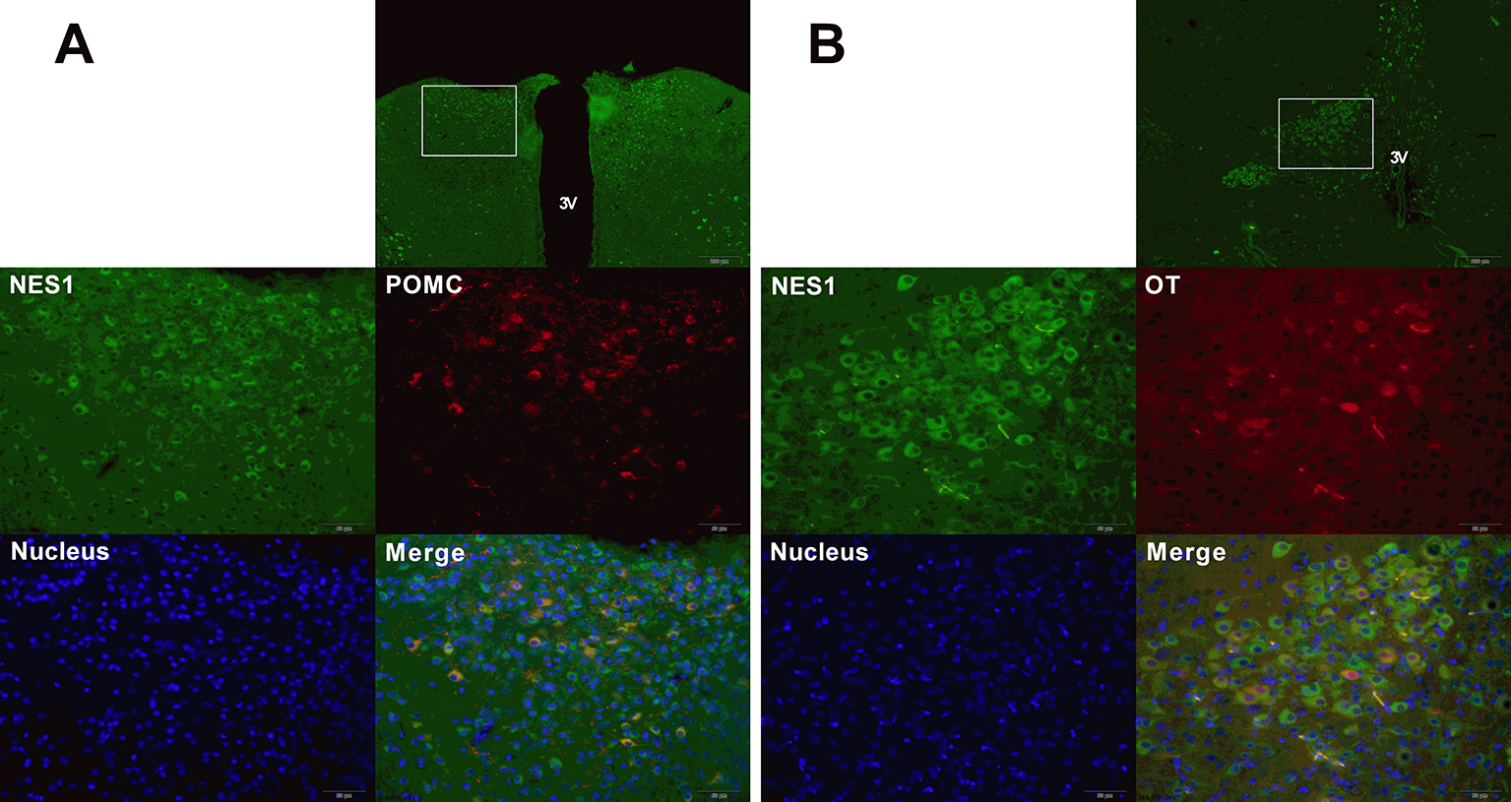
Co-expression of NES1 and POMC or OT in the rat hypothalamus. Hypothalamic sections were processed for double-fluorescence labeling for NES1 (green) and POMC/OT (red), and the nuclei were stained with DAPI (blue). **(A)** NES1 and POMC are co-expressed in the ARC. **(B)** NES1 and OT are co-expressed in the PVN. Micrographs in the first row of micrographs were captured at low magnification (scale bar = 200 μm, 100× magnification); the remaining micrographs were captured at high magnification (scale bar = 50 μm, 400× magnification); 3 V, third ventricle.

To determine the co-expression of NES1 and POMC (or OT) in the rat hypothalamus, hypothalamic sections were double labeled for immunofluorescence staining. As shown in **Figure 8**, NES1 staining, labeled with green fluorescence, was widely distributed in the form of granules in the ARC and PVN in the hypothalamus, while POMC and OT neurons in the hypothalamus were labeled with red fluorescence. POMC was only distributed in the ARC (**Figure 8A**), while OT was distributed in only the PVN (**Figure 8B**). Nuclei were stained blue by DAPI. In the ARC, the yellow sites indicate double-stained NES1 and POMC neurons, and in the PVN, the yellow sites indicate double-stained NES1 and OT neurons.

## Discussion

The focus of the current study was determining whether Xiaoyaosan attenuates depression-like behaviors and anorexia induced by CIS *via* regulation of the NES1-OT-POMC neural pathway in the hypothalamus. The main findings were as follows. (1) Xiaoyaosan improved CIS-induced depression-like behaviors and anorexia in rats. (2) Xiaoyaosan inhibited the increase in the levels of NES1 in the serum and hypothalamic PVN of rats after CIS. (3) Xiaoyaosan seemed to exert its antidepressant-like and anti-anorexia effects by regulating the NES1-OT-POMC neural pathway.

CIS models are often used in the study of depression, anxiety (30), cognitive deficits (31) and anorexia (32). Rats in this animal model show obvious depression-like behaviors and anorexia. The loss of appetite caused by long-term repeated stress is very similar to the pathogenesis of stagnation of liver qi and spleen deficiency syndrome in TCM (17). Our previous research indicated that Xiaoyaosan effectively regulates liver qi stagnation and spleen deficiency induced by CIS (24). Therefore, in the current study, we exposed rats to CIS for 21 days to study the antidepressant-like and anti-anorexia effects of Xiaoyaosan.

NES1 is a crucial peptide in stress-induced anorexia. The serum level of NES1 was significantly higher in rats after CIS than in control rats. Interestingly, the hypothalamic protein and mRNA expression levels of NES1 were not significantly different between the CIS group and the control group. Previously, 3 weeks of chronic stress has been shown to significantly increase hypothalamic NES1 expression (as shown by WB analysis) (33); however, another study indicated that 21 days of chronic stress did not increase hypothalamic NES1 mRNA expression (as shown by RT-qPCR) in rats (34). Because there are many different nuclei in the hypothalamus, NES1 neurons from these nuclei may show different sensitivities to different stress factors (8). We used immunohistochemistry, a widely used technique to detect the location of antigens in situ, to further investigate the expression of NES1 in the rat hypothalamus after CIS. After exposure to CIS, the expression of NES1-positive neurons in the PVN was significantly increased, while there was no significant difference in the expression of NES1-positive neurons in the ARC. Our observation that CIS upregulated NES1 expression in the PVN, which is correlated with depression-like conditions in rats, further confirms the pro-depressive role of NES1 (35).

The hypothalamic NES1-OT-POMC neural pathway plays an important role in regulating feeding behavior in the CNS (12, 14). In this study, rats exposed to CIS for 21 days exhibited activation of the hypothalamic NES1-OT-POMC neural pathway, and the expression of the NES1-related appetite suppressive neuropeptides OT, POMC, and MC4R was increased. In the PVN, OT neurons can be activated in situations of stress, leading to a loss of appetite (13). In the ARC, the melanocortin pathway, which is activated by stress, contributes to stress-induced anorexia (2, 36). Recently, POMC neurons in the ARC were shown to regulate long-term food intake (14). Double fluorescence experiments confirmed that NES1 and OT were co-expressed most extensively in the PVN, findings consistent with those of previous reports (12, 15).

Furthermore, OT neurons in the PVN are activated by exogenous (from the periphery) or endogenous (from the PVN) NES1. Central NES1 suppresses feeding in a melanocortin-dependent manner *via* the recruitment of OT signaling in the PVN (12), suggesting that NES1 is an upstream regulator of the appetite regulation pathway.

**Figure 9:**
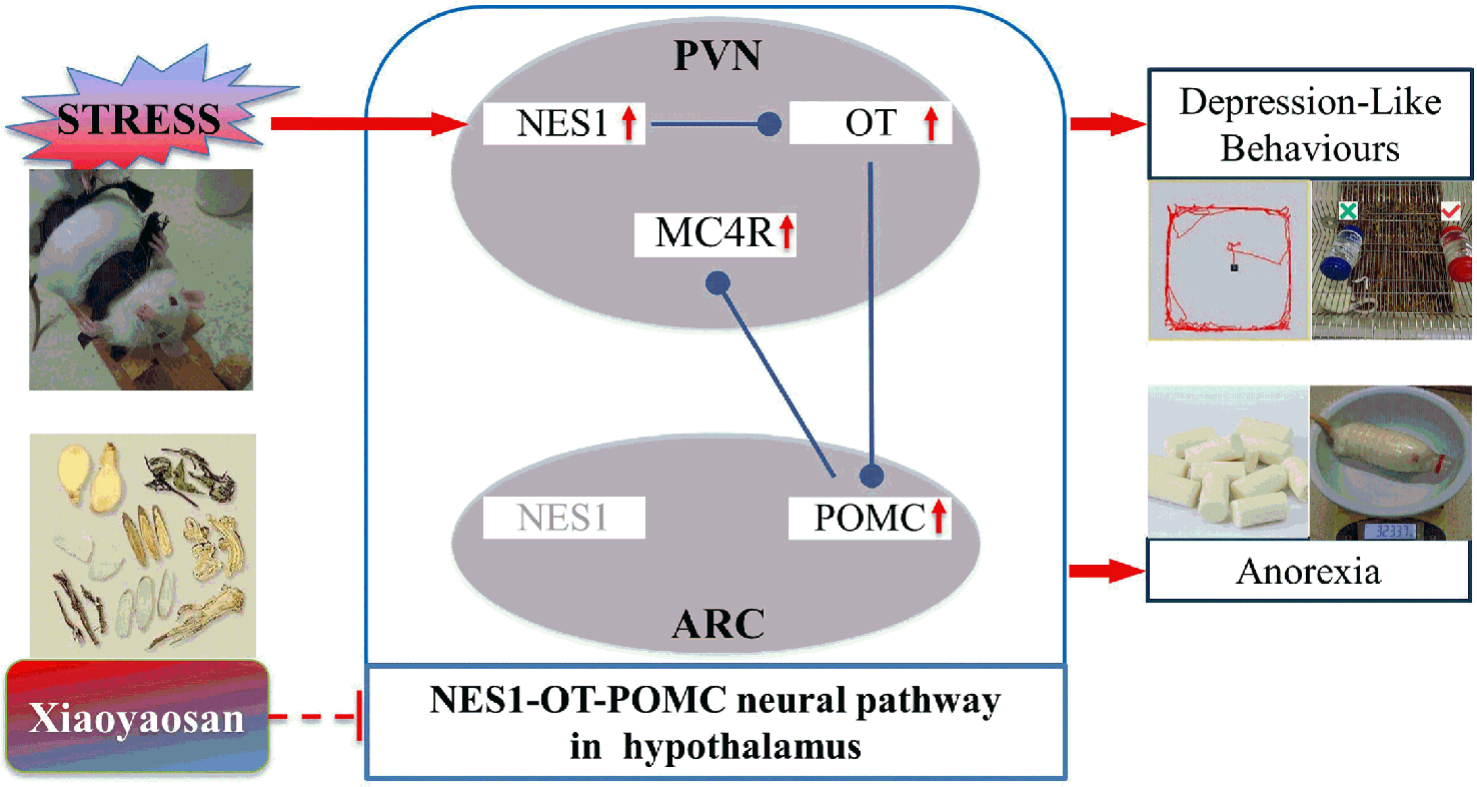
Potential mechanism by which Xiaoyaosan exerts antidepressant-like and anti-anorexia effects.

Xiaoyaosan is a popular medication used to treat both mental disorders and digestive system diseases (37, 38). In this experiment, CIS-induced depression-like behaviors and decreased food intake in rats were ameliorated by Xiaoyaosan, which is consistent with the results of our previous research (24). Furthermore, Xiaoyaosan treatment downregulated the expression of NES1 in both the serum and PVN, as shown by ELISA and immunohistochemistry. These observations suggest that Xiaoyaosan may exert its pharmacological effect by regulating NES1 signals from the periphery. Although the mechanism of this pharmacological effect remains unclear, several studies have shown that Xiaoyaosan improves depressive-like behavior through the modulation of peripheral organs, for instance, the liver (39) and the intestine (28, 40). Additionally, Xiaoyaosan downregulated the CIS-induced overexpression of OT, POMC, and MC4R in the rat hypothalamus. Therefore, Xiaoyaosan might partially inhibit the NES1-OT-POMC neural pathway and exert an antidepressant-like and anti-anorexia effects in this CIS model (**Figure 9**).

The widely used selective serotonin reuptake inhibitor (SSRI) fluoxetine is known to affect body weight and may help depressive patients improve their eating behavior (41). Fluoxetine treatment for 21 days has been shown to decrease the sensitivity of hypothalamic oxytocin-containing neurons (42). Fluoxetine also affects body weight by remodeling POMC neurons in the hypothalamus (43).

The present study has some limitations. First, we demonstrated an increase in the level of NES1 in the sera of CIS-exposed rats. However, the source of CIS-induced serum NES1 is not clear, indicating that the target of Xiaoyaosan among the peripheral organs is unknown. Second, the current study explains the antidepressant-like and anti-anorexia effects of Xiaoyaosan in the hypothalamus only, but whether Xiaoyaosan regulates the NES1 signal in the brain stem nucleus remains to be studied. Other studies have suggested that POMC neurons also project to the brainstem NTS, where they suppress appetite (15). Further studies using exogenous interventions, such as agonists (intraperitoneal injection of NES1) or blockers (injection of NES1 antibody), can be performed to explore the potential mechanism of Xiaoyaosan. Third, only male rats were included in this study. However, previous studies have suggested that dysfunction of NES1, which is involved in the pathology of some psychiatric disorders, might be sex specific (44). Therefore, further studies specifically designed to evaluate the antidepressant-like and anti-anorexia effects of Xiaoyaosan in different sexes should be performed.

## Conclusion

In conclusion, the current study evaluated the antidepressant-like and anti-anorexia effects of Xiaoyaosan in CIS-induced rats and found that the mechanism of these effects is related to regulation of the NES1-OT-POMC neural pathway. Our findings also provide new insight to further understand the pharmacological mechanism of Xiaoyaosan and alternative therapeutic approaches for the treatment of depression accompanied by a loss of appetite.

## Data Availability Statement

All datasets generated for this study are included in the article/**Supplementary Material**.

## Ethics Statement

The animal study was reviewed and approved by Institutional Animal Care and Use Committee at the Beijing University of Chinese Medicine.

## Author Contributions

QM and JC conceived and designed the experiments, QM and XL performed the research, and wrote the manuscript. ZY and HJ contributed to animal experiments. TW, YH, and YL contributed to conducted molecular experiments, QM, XL, and YJ analyzed the data, JC funded the research and revised the manuscript. All authors read and approved the final manuscript.

## Funding

This work was supported by the National Natural Science Foundation of China (No. 81803998, 81630104, 81973748), the Fundamental Research Funds for the Central Universities (China) (No. 21618334), and the Research Project of Traditional Chinese Medicine Bureau of Guangdong Province (No. 20191081).

## Conflict of Interest

The authors declare that the research was conducted in the absence of any commercial or financial relationships that could be construed as a potential conflict of interest.
