# Supplementary material for: Xiaoyaosan Ameliorates Chronic Immobilization Stress-Induced Depression-Like Behaviors and Anorexia in Rats: The Role of the Nesfatin-1–Oxytocin–Proopiomelanocortin Neural Pathway in the Hypothalamus
Source: Front Psychiatry. 2019 Dec 10;10:910. doi: 10.3389/fpsyt.2019.00910 (PMC6914835; doi:10.3389/fpsyt.2019.00910)
Supplement: Supplementary file 1 [file DataSheet_1.docx]

Supplementary material for:

Xiaoyaosan Ameliorates Chronic Immobilization Stress-Induced Depression-Like Behaviours and Anorexia in Rats: The Role of the Nesfatin-1-Oxytocin-Proopiomelanocortin Neural Pathway in the Hypothalamus

Qingyu Ma^1†^, Xiaojuan Li^1†^, Zhiyi Yan^2^, Haiyan Jiao^2^, Tingye Wang^2^, Yajing Hou^2^, Youming Jiang^2^, Yueyun Liu^2^ and Jiaxu Chen^1,2*^

^1^Formula-Pattern Research Center, School of Traditional Chinese Medicine, Jinan University, Guangzhou, Guangdong, China

^2^School of Traditional Chinese Medicine, Beijing University of Chinese Medicine, Beijing, China.

*** Correspondence:**Jiaxu Chen
chenjiaxu@hotmail.com

^†^These authors have contributed equally to this work.

**Supplement figure 1**





**Supplement figure 1.** The Kodak film displayed the multiple exposures of Fig 5E.

**Supplement figure 2**





**Supplement figure 2.** The Kodak film displayed the multiple exposures of Fig 5F.

**Supplement figure 3**





**Supplement figure 3.** The Kodak film displayed the multiple exposures of Fig 5G.
